# Predictive Validity of the Bayley-III Cognitive Scores at 6 Months for Cognitive Outcomes at 24 Months in Very-Low-Birth-Weight Infants

**DOI:** 10.3389/fped.2021.638449

**Published:** 2021-05-07

**Authors:** Tzu-Yu Liu, Jui-Hsing Chang, Chun-Chih Peng, Chyong-Hsin Hsu, Wai-Tim Jim, Jia-Ying Lin, Chia-Huei Chen, Sung-Tse Li, Hung-Yang Chang

**Affiliations:** ^1^Department of Pediatrics, Hsinchu MacKay Memorial Hospital, Hsinchu, Taiwan; ^2^Department of Pediatrics, MacKay Children's Hospital, Taipei, Taiwan; ^3^Department of Medicine, MacKay Medical College, New Taipei City, Taiwan; ^4^Premature Baby Foundation of Taiwan, Taipei, Taiwan

**Keywords:** very-low-birth-weight, cognitive function, neurodevelopment, Bayley-III, maternal socioeconomic status

## Abstract

**Purpose:** To assess the predictive validity of the Bayley Scales of Infant and Toddler Development, Third Edition (Bayley-III) cognitive scores at 6 months of corrected age (CA) for cognitive outcomes at 24 months of CA in very-low-birth-weight (VLBW) infants and investigate the predictors of change in cognitive outcomes.

**Methods:** We retrospectively evaluated VLBW children enrolled in the Taiwan Premature Infant Follow-up Network between 2010 and 2015 and completed the Bayley-III at CA of 6 and 24 months. The predictive validity of the cognitive performance at 6-month CA for the cognitive outcomes at 24-month CA was analyzed. The positive and negative predictive factors were also evaluated using logistic regression. Cut-off scores of <70 and <85 were used to identify lower functioning groups based on the Bayley-III definition.

**Results:** A total of 2,972 VLBW children, born with a mean weight of 1116.4 ± 257.5 g and mean gestational age of 29.0 ± 2.8 weeks, were evaluated. A cognitive score of <70 at 6-month CA had a positive predictive value (PPV) of 27.4% (95% confidence interval [CI]: 19.2–35.7%) for a cognitive score of <70 at 24-month CA, while the negative predictive value (NPV) was 97.3% (95% CI: 96.7–97.9%). A cut-off score of 85 had a PPV of 33.6% (95% CI: 28.1–39.0%) and an NPV of 87.7% (95% CI: 86.4–88.9%). Abnormal muscle tone at 6 months was a risk factor for cognitive function decline at 24 months for both Bayley-III cognitive cut-off scores: scores of 70 (adjusted odds ratio [AOR]: 2.8; 95% CI: 1.5–5.5) and 85 (AOR: 2.6; 95% CI: 1.6–4.1). Lower maternal socioeconomic status was associated with a worsening of the cognitive function in infants at 24 months who scored ≥85 at 6 months (AOR: 1.6; 95% CI: 1.2–2.0).

**Conclusion:** Subnormal Bayley-III cognitive scores at 6-month CA were not predictive of subnormal cognitive function at 24-month CA. In children with normal cognition during early infancy, abnormal muscle tone and lower maternal socioeconomic status may influence the cognitive developing process; this highlighted the importance of early identification of high risk infants and complete preterm infant-associated public health policies to promote an improved neurodevelopmental outcome.

## Introduction

Advances in perinatal and neonatal care have markedly improved the survival of very-low-birth-weight (VLBW, ≤1,500 g) infants (1–3). However, not only do the surviving VLBW infants experience high rates of short-term neonatal morbidities, but they are also at a high risk of long-term neurodevelopmental impairment (4). Cognitive dysfunction is the most common neurodevelopmental impairment in infants born with VLBW (5–7). Early intervention may improve cognitive and motor outcomes at infancy (8), highlighting the importance of regular neurodevelopmental monitoring and screening of VLBW infants after hospital discharge and the need for a reliable, sensitive screening tool to identify developmental deficits in early infancy.

The Bayley Scales of Infant Development has been widely used for the early identification and quantification of developmental delay and for determining eligibility for early intervention services in high-risk infants. However, the previous versions of Bayley Scales had limited therapeutic values because they did not delineate the different neurodevelopmental delays. It was difficult to discern cognitive impairments from language disabilities and to differentiate between gross and fine motor issues. Accordingly, the Bayley Scales of Infant and Toddler Development, 3rd Edition (Bayley-III) (9) has been updated to include index scores for cognitive, language, and motor domains.

Clinically, the Bayley-III is used to assess infants with a corrected age (CA) as young as 6 months. Although there have been studies that estimated the predictive validity of Bayley-III in early childhood (10–12), there are no data on the predictive value of the Bayley-III scores for preterm infants in early infancy. Impaired cognitive function is the most common neurologic complication in VLBW infants (5–7). Thus, the present study aimed to assess the predictive validity of the Bayley-III cognitive scales at a CA of 6 months for outcomes in VLBW children at a CA of 24 months. We also aimed to examine the correlates of the cognitive test scores between 6 and 24 months CA.

## Patients and Methods

### Study Design and Participants

This retrospective study was approved by the Mackay Memorial Hospital Institutional Review Board (approval no. 18MMHIS079). The need for informed consent was waived owing to the retrospective nature of the study. We evaluated VLBW children who were admitted to any of the 22 member hospitals of the Taiwan Premature Infant Follow-up Network between 2010 and 2015 and who had complete Bayley-III scales at a CA of 6 and 24 months. The network was funded by The Premature Baby Foundation of Taiwan. The exclusion criteria were infants with major congenital anomalies or heart disease. The infants were divided according to their cognitive scores at 24 months as detailed below.

### Program Protocol

The 22 member hospitals of the Taiwan Premature Infant Follow-up Network provide post-neonatal intensive care unit follow-up programs for those born with a VLBW (≤1,500 g). The case managers at each site collected parental consent and created case files for the VLBW infants admitted, including parental sociodemographic backgrounds, perinatal conditions, and medical histories in the neonatal intensive care unit. Demographic data included maternal age, marital status, level of education, and socioeconomic status. Parental education was categorized into seven levels based on academic degrees, and occupation was classified into eight classes according to job skills. Socioeconomic status, determined by education and occupation, was categorized into five classes (I–V), with class I representing the highest status and class V representing the lowest status.

Neonatal data included the presence of [1] bronchopulmonary dysplasia, defined according to the National Institute of Child Health and Human Development criteria (13); [2] severe intraventricular hemorrhage, defined as grades III and IV according to the criteria of Papile et al. (14); and [3] severe cranial ultrasound abnormalities during hospitalization, defined as congenital hydrocephalus, severe intraventricular hemorrhage, cystic periventricular leukomalacia, or persistent post hemorrhagic ventricular dilatation with or without any shunt placement at the time of discharge from hospital (15–20). Upon discharge, the infants are followed up at 6-, 12-, and 24-months CA. At each visit, the infants were evaluated clinically by a neonatologist and a certified psychologist using Bayley-III. Abnormal muscle tone was defined as spastic diplegia (bilateral paralysis of corresponding body parts), tetraparesis (weakness or paralysis of all four limbs), hemiparesis (partial unilateral paralysis), or hypotonia.

### Bayley-III

The Bayley-III generates scores for three composite indices (cognitive, language, and motor) and five subtests (cognitive, expressive communication, receptive communication, fine motor, and gross motor). In this study, we focused on the cognitive scale, which estimates general cognitive functioning based on non-verbal activities involving memory, problem solving, and manipulation. Age-standardized scores for each scale were calculated using test norms (mean, 100; standard deviation [SD], 15). Mild/moderate developmental delay was defined with scores of <-1 SD relative to the mean (scores <85), whereas moderate developmental delay was defined with scores of <-2 SDs relative to the mean (scores <70). Infants with developmental delay in any composite index were referred for early intervention during the follow-up.

### Statistical Analysis

The 6- and 24-month cognitive scores were compared using *t*-tests for continuous variables and chi-squared tests for categorical variables. We used cut-off scores of 70 and 85 to identify lower functioning groups based on the Bayley-III scores. The positive predictive value (PPV) i.e., the probability of a child having a cognitive score of <70 or <85 at 24 months, given a cognitive score of <70 or <85 at 6 months, and negative predictive value (NPV) i.e., the probability of having a cognitive score of ≥70 or ≥85 at 24 months, given a cognitive score of ≥70 or ≥85 at 6 months, were calculated as proportions for the total population of 2,972 children. Receiver operating characteristic (ROC) curves were constructed for mild/moderate and moderate delays at 24 months, and the area under the curve was calculated. To examine factors related to improvement and decline in cognitive scores from a CA of 6 months to a CA of 24 months, the children were divided into groups based on the cognitive scores at a CA of 24 months. Children with a cognitive score of <70 or ≥70 at a CA of 6 months were divided into two groups: those with a cognitive score of <70 and those with a cognitive score of ≥70 at a CA of 24 months. Similarly, children with cognitive scores of <85 or ≥85 at a CA of 6 months were also divided into two groups: those with cognitive scores of <85, which included those with scores of <70, and those with cognitive scores of ≥85 at a CA of 24 months. Logistic regression was subsequently used to examine factors associated with changes in cognitive outcomes across the two assessments for each of the two cut-off values. Additional predictors included in the analyses were sex, gestational age, and birth weight.

## Results

**Figure 1:**
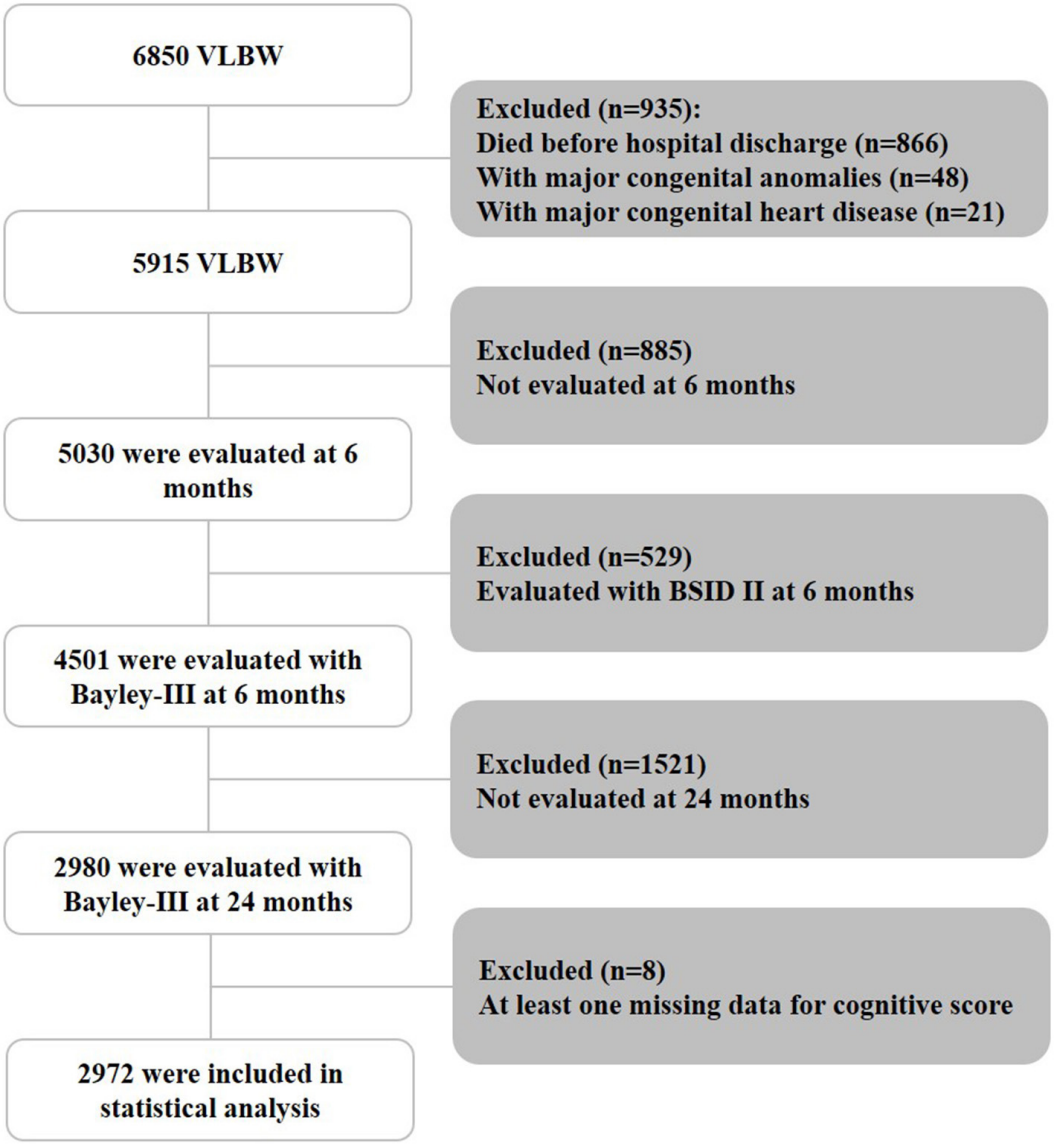
Patient inclusion flowchart. BSID II, Bayley Scales of Infant Development, Second Edition; VLBW, very low birth weight.

Of the 6,850 VLBW infants born between 2010 and 2015, 866 (12.6%) infants did not survive till hospital discharge, and 48 (0.7%) infants with major congenital anomalies and 21 (0.3%) infants with major congenital heart disease were excluded (Figure 1). Among the remaining 5,915 infants, 885 were not evaluated at 6 months, and 529 were evaluated using the Bayley-II assessment. In addition, 1,521 were lost to follow-up at 24 months. Eight children had at least one missing data point for the cognitive score. Thus, 2,972 infants who underwent the Bayley-III assessment at 6 and 24 months were included in the final analysis. There was no significant difference in the mean birth weight (1116.4 vs. 1122.1 g) or gestational age (29.0 vs. 29.0 weeks) between those assessed at 6- and 24- months CA and those who were only assessed at 6 months CA (*n* = 1,521). There were also no statistical differences in the rate of bronchopulmonary dysplasia (67.4 vs. 65.3%), necrotizing enterocolitis (5.4 vs. 5.7%), severe cranial ultrasound abnormalities (8.5 vs. 9.0%), average Bayley-III cognitive score (96.1 vs. 95.9), or the presence of abnormal muscle tone at a CA of 6 months (6.1 vs. 5.5%).

The mean cognitive score was 96.1 ± 12.8 at a CA of 6 months, and 93.8 ± 12.7 at a CA of 24 months. In total, 113 (3.8%) VLBW infants had a score of <70 at a CA of 6 months and 108 (3.63%) had a cognitive score of <70 at a CA of 24 months. There were 286 (9.6%) and 427 (14.4%) infants who had scores of <85 at a CA of 6 and 24 months, respectively (Table 1). Based on the neurodevelopment outcomes at 24 months CA, the sensitivity and specificity of 6-month cognitive score of <70 were 28.7% (95% confidence interval [CI]: 20.2–37.2%) and 97.1% (95% CI: 96.5–97.7%), respectively (Table 2). The PPV and NPV were 27.4% (95% CI: 19.2–35.7%) and 97.3% (95% CI: 96.7–97.9%), respectively. The cut-off score of <85 had a sensitivity of 22.5% (95% CI: 18.5–26.4%), specificity of 92.5% (95% CI: 91.5–93.6%), PPV of 33.6% (95% CI: 28.1–39.0%) and NPV of 87.7% (95% CI: 86.4–88.9). Cutoff values of the Bayley III cognitive score at 6 months based on the ROC analysis were 87.5 (sensitivity, 40.7%; specificity, 79.3%; area under the curve, 0.629 [95% CI: 0.598–0.659]) and 82.5 (sensitivity, 44.4%; specificity, 91.7%; area under the curve, 0.715 [95% CI, 0.656–0.775]) in predicting mild/moderate and moderate cognitive delay, respectively.

**Table 1:** Rates of moderate delay (<70), mild delay (70–84), and normal cognitive scores (≥ 85) at 6 and 24 months of corrected age in 2,972 patients.

**Bayley-III cognitive scale score**				
	**24 months**			**Total, n (%)**
6 months	< 70	70–84	≥ 85	
< 70	31	19	63	113 (3.8%)
70–84	17	29	127	173 (5.8%)
≥85	60	271	2,355	2,686 (90.4%)
Total, n (%)	108 (3.6%)	319 (10.8%)	2,545 (85.6%)	2,972

**Table 2:** Sensitivity, specificity, positive, and negative predictive values for Bayley-III cognitive score at 6 months at predicting 24-month outcomes in moderate delay (<70) and mild/moderate delay (<85).

	**Moderate delay (<70)**	**Mild/moderate delay (<85)**
Sensitivity	28.7% (20.2–37.2%)	22.5% (18.5–26.4%)
Specificity	97.1% (96.5–97.7%)	92.5% (91.5–93.6%)
Positive predictive value	27.4% (19.2–35.7%)	33.6% (28.1–39.0%)
Negative predictive value	97.3% (96.7–97.9%)	87.7% (86.4–88.9%)

Univariate comparisons of maternal sociodemographic, birth, and neonatal data of children whose cognitive scores improved or decreased from a CA of 6- to 24- months from each cut-off score (70 and 85) and those who were in the subnormal category are presented in Tables 3, 4. Compared with children with subnormal scores at both time points, those who scored <70 at 6 months and improved to ≥70 at 24 months had significantly lower rates of sepsis and severe cranial ultrasound abnormalities during hospitalization, and lower rates of abnormal muscle tone at a CA of 6 months. There were no significant between-group differences in other sociodemographic factors including maternal marital status, education, or socioeconomic status; and any birth data such as sex and rate of extremely preterm (gestational age, <28 weeks) or extremely low birth weight (≤1,000 g). Compared with children whose score increased to ≥85, those whose scores remained <85 had a significantly higher rate of extremely low birth weight, severe cranial ultrasound abnormalities during hospitalization, and abnormal muscle tone at a CA of 6 months.

**Table 3:** Comparison of maternal demographic and infant data according to change in cognitive scores from 6 to 24 months in cut-off score of 70.

**6 months**	**Cognitive score <70**		**Cognitive score ≥70**	
**24 months**	**Cognitive score <70** **(*n* = 31)**	**Cognitive score ≥70** **(*n* = 82)**	**Cognitive score <70** **(*n* = 77)**	**Cognitive score ≥70** **(*n* = 2,782)**
**Maternal demographic data, n (%)**				
Married	29/31 (93.5%)	76/81 (93.8%)	75/77 (97.4%)	2,660/2,773 (95.9%)
**Education**				
High school or higher	28/30 (93.3%)	73/77 (94.8%)	70/76 (92.1%)	2,601/2,715 (95.8%)
College or higher	18/30 (60.0%)	45/77 (58.4%)	47/76 (61.8%)	1,922/2,715 (70.8%)
**Socioeconomic status**				
Class IV or V	14/30 (46.7%)	34/76 (44.7%)	31/75 (41.3%)	899/2,696 (33.3%)
Class V	4/30 (13.3%)	5/76 (6.6%)	7/75 (9.3%)	148/2,696 (5.5%)
**Infant data, n (%)**				
Gestational age <28 weeks	16/31 (51.6%)	34/81 (42.0%)	33/77 (42.9%)	845/2,781 (30.4%)^*^
Birth weight ≤1,000 g	21/31 (67.7%)	45/81 (55.6%)	38/77 (49.4%)	901/2,781 (32.4%)^**^
Sex, male	21/31 (67.7%)	49/81 (60.5%)	39/77 (50.6%)	1,388/2,781 (49.9%)
Sepsis	17/31 (54.8%)	17/81 (21.0%)^**^	14/77 (18.2%)	449/2,781 (16.1%)
Necrotizing enterocolitis	4/31 (12.9%)	10/82 (12.2%)	5/77 (6.5%)	143/2,781 (5.1%)
Bronchopulmonary dysplasia	28/31 (90.3%)	61/80 (76.3%)	48/76 (63.2%)	1,859/2,777 (66.9%)
Severely abnormal cerebral ultrasound	13/31 (41.9%)	16/81 (19.8%)^*^	18/77 (23.4%)	193/2,779 (6.9%)^***^
Abnormal muscle tone at 6 months	15/30 (50.0%)	22/82 (26.8%)^*^	13/77 (16.9%)	129/2,774 (4.7%)^***^
Any early intervention referral at 6 months	23/29 (79.3%)	55/81 (67.9%)	29/76 (38.2%)	664/2,693 (24.7%)^**^

*There were missing data in some parameters; the number at the bottom states the overall number of available data*.

* *p < 0.05*,

** *p < 0.01*,

*** *p < 0.001*.

**Table 4:** Comparison of maternal demographic and infant data according to change in cognitive scores from 6 to 24 months in cut-off score of 85.

**6 months**	**Cognitive score <85**		**Cognitive score ≥85**	
**24 months**	**Cognitive score <85** **(*n* = 96)**	**Cognitive score ≥85** **(*n* = 190)**	**Cognitive score <85** **(*n* = 331)**	**Cognitive score ≥85** **(*n* = 2,355)**
**Maternal demographic data, n (%)**				
Married	90/96 (93.8%)	177/189 (93.7%)	311/331 (94.0%)	2,262/2,346 (96.4%)^*^
**Education**				
High school or higher	87/92 (94.6%)	173/182 (95.1%)	305/324 (94.1%)	2,207/2,300 (96.0%)
College or higher	51/92 (55.4%)	121/182 (66.5%)	198/324 (61.1%)	1,662/2,300 (72.3%)^***^
**Socioeconomic status**				
Class IV or V	44/91 (48.4%)	67/181 (37.0%)	139/322 (43.2%)	728/2,283 (31.9%)^***^
Class V	8/91 (8.8%)	11/181 (6.1%)	24/322 (7.5%)	121/2,283 (5.3%)
**Infant data, n (%)**				
Gestational age <28 weeks	47/96 (49.0%)	73/189 (38.6%)	105/331 (31.7%)	703/2,354 (29.9%)
Birth weight ≤1,000 g	60/96 (62.5%)	86/189 (45.5%)^**^	111/331 (33.5%)	748/2,354 (31.8%)
Sex, male	55/96 (57.3%)	112/189 (59.3%)	169/331 (51.1%)	1,161/2,354 (49.3%)
Sepsis	28/96 (29.2%)	37/189 (19.6%)	53/331 (16.0%)	379/2,354 (16.1%)
Necrotizing enterocolitis	13/96 (13.5%)	13/189 (6.9%)	20/331 (6.0%)	116/2,354 (4.9%)
Bronchopulmonary dysplasia	78/95 (82.1%)	142/188 (75.5%)	216/331 (65.3%)	1,560/2,350 (66.4%)
Severely abnormal cerebral ultrasound	33/96 (34.4%)	31/189 (16.4%)^**^	33/331 (10.0%)	143/2,352 (6.1%)^**^
Abnormal muscle tone at 6 months	34/95 (35.8%)	37/188 (19.7%)^**^	29/331 (8.8%)	79/2,349 (3.4%)^***^
Any early intervention referral at 6 months	71/94 (75.5%)	112/188 (59.6%)^**^	85/318 (26.7%)	503/2,279 (22.1%)

*There were missing data in some parameters; the number at the bottom states the overall number of available data*.

* *p < 0.05*,

** *p < 0.01*,

*** *p < 0.001*.

Children who declined at 24 months, with a cognitive score of ≥70 at 6 months and <70 at 24 months, were more likely to be extremely preterm, to have an extremely low birth weight, severe cranial ultrasound abnormalities during hospitalization, abnormal muscle tone at a CA of 6 months, and higher rate of any early intervention referral at 6 months. The same characteristics were also observed in the group with a cognitive score of <85 at 6 months remained <85 at 24 months. In the group that had a cut-off score of 85, sociodemographic factors including maternal education and socioeconomic status were also correlated to the predictive validity of the cognitive scores at 6 months CA. Specifically, the rate of cognitive score that decreased to <85 at 24 months was significantly higher in children whose mothers had lower level of education and socioeconomic status. Children whose cognitive scores remained unchanged had significantly lower rates of severe cranial ultrasound abnormalities during hospitalization and abnormal muscle tone at a CA of 6 months.

Logistic regression analyses adjusted for sex, gestational age, and birth weight showed that severe cranial ultrasound abnormalities during hospitalization was a risk factor for the lack of cognitive improvement both from <70 to ≥70 (adjusted odds ratio [AOR]: 3.3; 95% CI: 1.1–9.7; *p* < 0.05) and <85 to ≥85 (AOR: 2.1; 95% CI: 1.2–4.0; *p* < 0.05); in other words, children with subnormal cognitive scores at 6 months remained subnormal at 24 months. Abnormal muscle tone at 6 months was also a risk factor for both adverse cognitive changes from ≥70 to <70 (AOR: 2.8; 95% CI: 1.5–5.5; *p* < 0.01), and ≥85 to <85 (AOR: 2.6; 95% CI: 1.6–4.1; *p* < 0.001) at 24 months. Notably, in the subgroup analysis of those with normal cognitive scores (≥85) at 6 months, a lower maternal socioeconomic status (class IV or V) was a risk factor for a decline to <85 at 24 months (AOR: 1.6; 95% CI: 1.2–2.0; *p* < 0.001).

## Discussion

There are limited data on the predictive value of Bayley-III scores for preterm infants in early infancy. In this study, we evaluated the predictive validity of 6 months CA cognitive Bayley-III scores for the cognitive function of VLBW infants at 24 months CA. We showed that a subnormal Bayley-III score at a CA of 6 months in VLBW infants had a high NPV and specificity for cognitive impairment at a CA of 24 months. To the best of our best knowledge, this is the largest cohort study to report the predictive validity of the Bayley-III cognitive score in VLBW infants within a CA of 2 years.

Most cognitively normal infants continue to have normal cognitive development as toddlers. However, subnormal Bayley-III cognitive scores at a 6-month CA had low PPV for the cognitive scores at 24 months CA. In other words, children with suboptimal cognitive performance at 6 months CA may still have normal cognitive function at 24 months. This is a reasonable result because cognitive development is affected by various biological and psychosocial factors through parent-infant interactions (21) and positive early interaction patterns (8). While the predictive power of Bayley-III in older age groups has already been demonstrated, few studies have evaluated the predictive validity of the Bayley-III cognitive score within a CA of 2 years. Bode et al. (10) reported a high correlation between Bayley-III cognitive scores at 2 years of age and subsequent intelligence quotient at 4 years of age in extremely preterm infants. Compared with our results, they reported a higher sensitivity and PPV (85 and 64%, respectively), lower specificity (87%), and similar NPV (95%) by using mild/moderate delay classification. They also had a higher sensitivity and PPV (71 and 71%, respectively), and a similar level of specificity and NPV (96 and 96%, respectively), by using moderate delay classification. Spencer-Smith et al. (12) surveyed a similar population and showed that the Bayley-III cognitive score was positively associated with later cognitive functioning, but a Bayley-III cognitive score indicative of mild/moderate delay at 24 months of age was not strongly predictive of a cognitive impairment at 4 years of age, with a low sensitivity and PPV (29 and 50%, respectively), and good specificity and NPV (94 and 87%, respectively). Although the PPV of Bayley III differed in the two studies, they both showed that the cognitive score of Bayley-III has a high NPV in preterm infants when used for prediction in older age groups.

In our study, we found that severe cranial ultrasound abnormalities during hospitalization was a primary factor associated with adverse cognitive development. Hack et al. (22) reported similar findings in that a severely abnormal cranial sonography result was associated with a lack of improvement in cognitive function. The adverse effects of severely abnormal cranial sonography results on long-term neurodevelopmental outcomes are well-identified (17–19, 23–25). It is reasonable to assume that children with severely abnormal cranial sonography scans will have chronic subnormal cognitive function. We also discovered that children with a history of severe abnormal cranial findings had a higher risk of abnormal cognitive function at a CA of 24 months irrespective of their performance at 6 months CA. A similar adverse effect was also seen in children presenting an abnormal muscle tone at a CA of 6 months. Abnormal muscle tone is a major feature of cerebral palsy, which is one of the main factors associated with poor neurodevelopmental outcomes (25, 26). In general, children with cerebral palsy have some level or a greater likelihood of developing cognitive impairments. However, a diagnosis of cerebral palsy in early infancy is challenging. Therefore, extra care should be taken to diagnose cerebral palsy in infancy. Premature infants often have varying degrees of abnormal muscle tone. The motions that can be performed at 6 months of CA are also limited and difficult to evaluate, which may result in fluctuating results at evaluation at 24 months of CA. In previous studies, it was noted that many tone abnormalities detected at 6 months may resolve by 12 months of age (27). In our follow-up, only 24% of children with abnormal muscle tone at a CA of 6 months presented cerebral palsy at a CA of 24 months. However, we did not aim to diagnose cerebral palsy at an early stage but to identify children at risk of experiencing adverse changes in cognitive function during early childhood. The neuroanatomical pathway of these adverse cognitive function changes is unclear; however, the affected parts of infant brains are probably associated with cognition. Furthermore, impaired motor function in children with severe brain injuries would negatively affect their performance during an assessment with the Bayley cognitive scales.

We also found a significant effect of maternal socioeconomic status on cognitive development. Of the 2,686 (90.4%) children with normal cognitive scores (≥85) at a CA of 6 months in our cohort, 278 (10.3%) children had adverse changes in cognitive scores (<85) at a CA of 24 months without any severe cranial ultrasound abnormalities during hospitalization or abnormal muscle tone noted at a CA of 6 months. Lower maternal socioeconomic status was associated with this adverse change, and it highlights the importance of promoting government policies to support families with low socioeconomic status with VLBW infants, whether through financial assistance or strengthening childcare instruction, to reduce the gap caused by socioeconomic status. This finding is also important as it supports the strong association between maternal socioeconomic status and neurodevelopmental outcomes previously described (28). Higher maternal socioeconomic status may have an association with better developmental outcomes (29). However, our findings did not show that a better maternal socioeconomic status had an advantageous effect in those with subnormal cognitive function in early infancy. Conversely, we found that lower maternal socioeconomic status may have an adverse effect on children with normal cognitive function at a CA of 6 months. This is consistent with the report that socioeconomic status has a less profound impact on children with a higher biological risk of neurodevelopment outcomes (30). McDermott et al. recently reported associations between parental socioeconomic status and both region-specific brain morphology and cognitive outcomes, which may elucidate potential neuroanatomical mediators (31). Additional studies are needed to determine if socioeconomic status in childhood exerts a deterministic effect on cognitive development.

Our study is limited by its retrospective nature. The follow-up rate might cause a bias possibility. Only 66.2% of the infants who completed the Bayley-III assessment at a CA of 6 months were also assessed at 24 months, when ideally it should have been over 80%. Although there were no characteristic differences, the socioeconomic status of mothers was significantly lower in those who were lost to follow-up, which is similar to results of a previous study (2). Furthermore, there is no difference in the proportion of infants with scores of <70 at a CA of 6 months between those lost to follow-up and those who completed follow-up. However, the proportion of infants with scores of ≥85 at a CA of 6 months was lower in the group lost to follow-up (87.6 vs. 90.4%). It is, therefore, difficult to speculate on the possible bias introduced by infants who were lost to follow-up in this study. In a previous systemic review, early developmental interventions for preterm infants had a positive influence on cognitive outcomes during infancy until preschool age (8). It was difficult to demonstrate this positive effect in this study as only 26.8% of the overall cohort received an early intervention (61.0 and 70.9% of those with mild/moderate and moderate delay at a CA of 6 months, respectively). These rates were similar or even higher than those in other reports (32, 33). Although higher rate of early intervention referral was associated with worse outcomes in some groups, the causal relationship between early intervention and neurodevelopmental outcomes could not be established based on this association. There were no standard criteria for referral to an early intervention in our follow-up protocol. Physicians referred any infants with potential developmental delays or with subnormal Bayley-III scale scores depending on the patient's clinical conditions. The CA at the start of the interventions also varied in each case. High risk infants were referred to specialists for early interventions right after hospital discharge. After the first neurodevelopmental assessment was done at 6 months of CA, those showing subnormal Bayley-III scores or abnormal physical findings without being in the process of early interventions were referred immediately. These factors may have influenced the effect of the interventions in our study. The effects of a standardized early developmental intervention in patients with neurodevelopmental impairment should be further investigated. Developing different models of intervention programs at an early age for infants at a high risk of neurodevelopmental problems for prevention, remediation, or treatment of a specific delay or disability, in the future, may be helpful for further research. Evidence also suggests that early high-quality parent-infant interactions could positively in?uence cognitive and social development in children. Promotion of parent-children reading activities may provide an additional benefit to those with neurodevelopmental impairment during infancy. This study only focused on the predictive validity of the Bayley-II cognitive score owing to the scope of the topic. However, it is generally agreed that there are correlations among motor skills, cognition, and language with regards to neurodevelopment. Further analysis of all three composite scores of the Bayley-III scale and the identification of issues in different domains may potentially improve the predictive value for developmental delay. It has been shown that the US norms of the Bayley-III might be not adequate in other populations. Yu et al. recommended an upward adjustment of Bayley-III cognitive scores for moderate developmental delay in Taiwanese infants which included scores of 80 at 6 months and 90 at 24 months, rather than 70 (34). However, Yu et al. did not demonstrate the Bayley-III adjusted cut-off scores for mild/moderate delay in Taiwanese infants. In the Taiwan Premature Infant Follow-up Network, mild/moderate delay and moderated delay are still defined as composite scores of <85 and <70, respectively, in agreement with US norms. Nevertheless, using the scores recommended by Yu et al. did not improve the predictive validity. Based on the Bayley-III cognitive scores of <90 at 24 months, the sensitivity and specificity of the 6-month cognitive scores of <80 were 14.0% (95% CI: 11.4–16.6%) and 95.2% (95% CI: 94.3–96.0%), respectively. The PPV and NPV were 46.1% (95% CI: 39.3–52.9%) and 78.9% (95% CI: 77.3–80.4%), respectively. Regarding the Taiwanese norm for Bayley-III, further investigation is required.

In conclusion, the Bayley-III cognitive score at a CA of 6 months cannot reliably predict the cognitive dysfunction at a CA of 24 months. However, there was no other scoring system that was as widely used and researched as the Bayley Scales of Infant and Toddler Development for the identification and quantification of developmental delay as early as at 6 months of age. VLBW infants still need to begin neurodevelopment assessment as early as a CA of 6 months to identify those at risk of later cognitive impairment. This allows high-risk infants to begin intervention programs for preventing further delay, thereby compensating for deficits to promote optimal function and independence. This is particularly important for those with any severe cranial ultrasound abnormalities during hospitalization, abnormal muscle tone presentation in early infancy, and/or those with a lower maternal socioeconomic status. Finally, preterm infant-associated public health policies should provide more financial assistance or preemie-care instruction to promote better neurodevelopmental outcomes.

## Data Availability Statement

The data analyzed in this study is subject to the following licenses/restrictions: The data that support the findings of this study are available from the Premature Baby Foundation of Taiwan. Restrictions apply to the availability of these data, which were used under license for this study. Data are not available without the permission of the Premature Baby Foundation of Taiwan. Requests to access these datasets should be directed to Premature Baby Foundation of Taiwan, pbf@pbf.org.tw.

## Ethics Statement

The studies involving human participants were reviewed and approved by Mackay Memorial Hospital Institutional Review Board. Written informed consent from the participants' legal guardian/next of kin was not required to participate in this study in accordance with the national legislation and the institutional requirements.

## Author Contributions

All authors listed have made a substantial, direct, and intellectual contribution to the work and approved it for publication.

## Conflict of Interest

The authors declare that the research was conducted in the absence of any commercial or financial relationships that could be construed as a potential conflict of interest.
